# Henoch Schonlein Purpura – A 5-Year Review and Proposed Pathway

**DOI:** 10.1371/journal.pone.0029512

**Published:** 2012-01-03

**Authors:** Louise Watson, Amanda R. W. Richardson, Richard C. L. Holt, Caroline A. Jones, Michael W. Beresford

**Affiliations:** 1 Department of Women's and Children's Health, Institute of Translational Medicine, University of Liverpool, Alder Hey Children's NHS Foundation Trust Hospital, Liverpool, United Kingdom; 2 Department of Paediatric Nephrology, Alder Hey Children's NHS Foundation Trust Hospital, Liverpool, United Kingdom; Murdoch Childrens Research Institute, Australia

## Abstract

Henoch Schonlein Purpura (HSP) is the commonest systemic vasculitis of childhood typically presenting with a palpable purpuric rash and frequently involving the renal system. We are the first group to clinically assess, critically analyse and subsequently revise a nurse led monitoring pathway for this condition.

A cohort of 102 children presenting with HSP to a secondary/tertiary level UK paediatric hospital over a five year period, were monitored using a nurse led care pathway. Using this cohort, the incidence (6.21 cases per 100,000 children per year) and natural disease course of HSP nephritis (46% initial renal inflammation; 9% subsequent renal referral; 1% renal biopsy and immunosuppression) was determined. Older patients were at higher risk of requiring a renal referral (renal referral 12.3 (8.4–13.5) years vs. normal outcome 6.0 (3.7–8.5) years; p<0.01). A normal urinalysis on day 7 had a 97% (confidence interval 90 to 99%) negative predictive value in predicting a normal renal outcome.

Using this data and existing literature base, The Alder Hey Henoch Schonlein Purpura Pathway was developed, a revised pathway for the screening of poor renal outcome in HSP. This is based on a six-month monitoring period for all patients presenting with HSP, which importantly prioritises patients according to the urine findings on day 7 and thus intensively monitors those at higher risk of developing nephritis. The pathway could be easily adapted for use in different settings and resources.

The introduction of a standardised pathway for the monitoring of HSP will facilitate the implementation of disease registries to further our understanding of the condition and permit future clinical trials.

## Introduction

Henoch Schonlein Purpura (HSP) is the commonest systemic vasculitis of childhood with a reported incidence of 10–20 cases per 100,000 children per year [1]. HSP can present at any age, but is most common in children under five. Generally the prognosis is good, with the exception of those with significant renal involvement [2].

The principal aim of HSP follow up is detecting persistent renal inflammation, which undiagnosed, could progress to permanent renal damage [3]. Due to the asymptomatic nature of HSP associated nephritis, unlike all other associated complications, most centres provide a program of regular urine and blood pressure (BP) monitoring for up to 12 months. The long term risk of permanent renal impairment in patients with minor urine abnormalities is low (1.6% reported previously [2]). However, this rises to 19.5% in children with nephrotic or nephritic features [2], [4]. A meta-analysis on the use of early corticosteroids in this condition suggested some effect on the renal outcome [5] and immunosuppressive treatments are used in severe cases, as they are believed to preserve renal function, despite a paucity of randomised controlled trials. Universal follow up for patients with HSP imposes a financial burden on healthcare services and increases parental anxiety and inconvenience. Its role remains uncertain and there is need for standardised evidence based monitoring.

A nurse led HSP renal monitoring care pathway has been implemented at Alder Hey Children's Hospital (UK) for over five years. The first aim of this study was to describe the disease course in a cohort of patients on this pathway, recording the occurrence of proteinuria and the resolution of renal inflammation. The second aim, using these data and previous published cohort data, was to develop a revised pathway for the screening of HSP associated nephritis.

## Results

### Subjects

**Figure 1:**
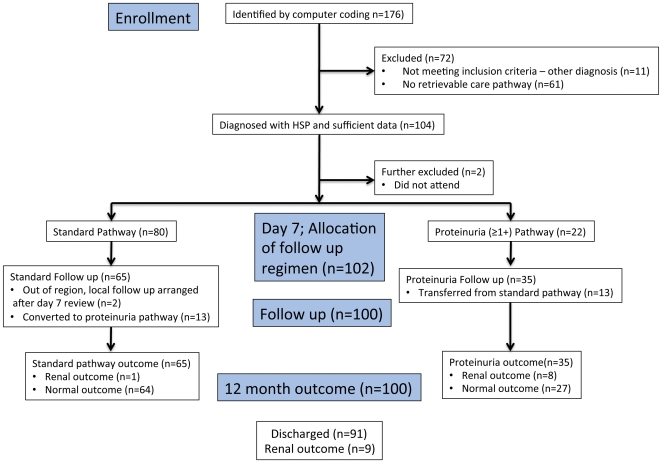
The HSP cohort - recruitment, follow up and outcome.

Using the hospital coding system, 176 patients were identified during June 2004 to February 2010, a 5.67-year period. Following case note inspection 165 patients had a diagnosis of HSP, 61 patients were ineligible for further analysis, as the pathway data was not available. Using the 165 cases presenting over 5.67 years, the calculated incidence was 6.21 HSP cases per 100,000 Liverpool children (aged 0–15 years), per year (Source: 2001 UK Census: Standard Area Statistics (England and Wales)). Figure 1 outlines the recruitment and subsequent follow up for the study cohort.

The median age was 6.3 (range 0.3–15.4; IQR 4.0–9.5) years with 48% male gender and 90% Caucasian ethnicity. The median length of follow up was 12 months. Those requiring renal referral were significantly older than those with a normal renal outcome (renal referral 12.3 (8.4–13.5) years vs. normal outcome 6.0 (3.7–8.5) years; p<0.01).

### Presenting features

Of 102 patients where pathway review took place, presenting symptoms included: palpable purpuric rash (100%), arthralgia (45%), joint swelling (19%), abdominal pain (9%), fever (5%), lethargy (1%), bloody stools (1%), haematuria (1%), scrotal pain (1%) and limp (1%). No patients were nephrotic, nephritic or had an abnormal renal function at the initial diagnosis. Two patients presented with a second recurrence of HSP with the first episode being uncomplicated and before pathway implementation.

### Hypertension

At diagnosis/presentation, 14% of patients were hypertensive, including 10% with a BP >99^th^ centile. This hypertension was unlikely recognised as it did not prompt further evaluation, 64% of patients who presented with hypertension had a normal urinalysis, 28% had proteinuria (≥1+) and one patient (7%) had mixed proteinuria (1+) and haematuria (3+). Importantly, there were no patient episodes of hypertension after entry to the follow up pathway (day 7) in any child, including those who required medical review.

### Initial Urinalysis

At diagnosis 53% (55/102) had a normal urinalysis, 37% had isolated proteinuria, 2% had isolated haematuria (one macroscopic) and 7% had mixed proteinuria and haematuria. 1 patient had no urine result documented.

### Monitoring pathway

At day 7 review, patients were triaged into follow up regimens, 80 patients started on the standard pathway, as they had no evidence of proteinuria at that time and 22 patients on the proteinuria pathway. After 12 months follow up 13 patients developed proteinuria after day 7, giving a total of 35 patients on the proteinuria pathway. Overall 91 patients could be discharged after 12 months and 9 patients were (or had already been) referred to a paediatrician in view of persistent urine abnormalities or meeting the exit criteria.

### Normal urinalysis at presentation

Patients with a normal urinalysis at day 7 but who later transferred to the proteinuria pathway (n = 13) developed abnormalities up to 6 months (day 168). In all patients presenting with the first occurrence of an abnormal urinalysis after 1 month (day 28), the proteinuria (1+) was an isolated episode with a normal urine albumin:creatinine ratio and a normal outcome. Within the group of patients with a normal urinalysis at day 7, two required a renal referral, one of which transferred to the proteinuria pathway before day 28, described in the renal outcome section below, the second patient was referred at 12 months for persistent microscopic haematuria (1+), which resolved by 14 months.

### Renal outcome

Nine patients from the study group met the primary outcome of a medical referral, two of these had an early rise in UACR and were referred within 3 months from diagnosis for nephrology review (one of which started on the standard pathway described above); the other seven patients had persistent proteinuria or haematuria at 12 months. Four of these with persistent changes remain under nephrology follow up with on going microscopic urine abnormalities but normal renal function and normal blood pressure. Out of the two patients with a raised urine protein level exiting the pathway early, one developed nephrotic syndrome within the first month following diagnosis and a renal biopsy revealed grade III HSP nephritis according to the ISN classification [6]; they required immunosuppression, angiotensin converting enzyme (ACE) inhibition and a repeat biopsy seven months later demonstrated improvement with grade II HSP nephritis. They remain under nephrology review on ACE inhibition with microscopic haematuria and proteinuria, but with normal renal function and normotensive. The second patient had a peak UACR of 408 mg/mmol at day 14, was referred to a nephrologist with resolution of proteinuria over the subsequent month. He remains under observation with a normal urinalysis and normal blood pressure.

### Positive and negative predictive values

Using the urinalysis results from day 7, the positive and negative predictive values and sensitivity and specificity for a medical referral were calculated (table 1). The positive predictive value of the finding of proteinuria on urinalysis testing at day 7 and a renal outcome was 32% (15 to 55%) and the negative predictive value was 97% (90 to 99%) [7], [8]. The sensitivity of the day seven urinalysis and the need for a medical referral was 77.8% (45.3–93.7%) and specificity 83.5% (74.6–89.8%).

**Table 1:** The urinalysis (day 7) and the occurrence of a renal outcome.

	Proteinuria on urinalysis	No protein on urinalysis	Total
Clinician referral	7 (True positive)	2 (False negative)	9
Discharged	15(False positive)	76 (True positive)	91
Total	22	78	100

Each patient diagnosed with HSP had a random urinalysis taken and subsequent monitoring frequency determined on the presence or absence of proteinuria.

### Other clinical outcomes

Two patients required a rheumatology referral due to a persistent purpuric rash, but the diagnosis was upheld.

### Patients with missing pathway data

The hospital computerised appointment and results system was searched for evidence of renal histology, reviews and abnormal urinalysis or blood tests, for those patients with irretrievable pathway data. There were no significant differences between the group with missing pathway data and the study cohort with regards to demographic data (age p = 0.157, sex p = 0.67, ethnicity p = 0.90) or renal outcome (p = 0.055). From the 61 patients excluded, one patient had undergone a renal biopsy revealing grade III HSP nephropathy and two patients had seen a nephrologist with recent discharge from follow up. The exact details of these patients were unavailable and further conclusions could not be established.

## Discussion

This is the first study to our knowledge, to clinically evaluate, critically appraise, and subsequently revise, a care pathway for the monitoring of HSP nephritis. Our data, together with other studies, demonstrates an overall low risk of HSP associated nephritis in an unselected cohort of patients presenting with features of HSP, 91% had a self-limiting disease course in our series. There have now been several cohorts describing this, together with the finding that patients with HSP who present with or develop proteinuria are at the greatest risk of renal complications [2], [3], [4], [9], [10], [11], [12], [13], [14], [15], [16], [17], [18]. A stratified approach to monitoring which is robust enough to allow early identification of the high-risk patients balanced against the inconvenience to the family, resource implications and burden to healthcare providers of prolonged or costly follow up therefore seems appropriate.

The first aim of our study was to evaluate the rate of renal involvement using a nurse led monitoring pathway. Our cohort identified renal involvement in 46% of patients at presentation with an eventual 9% requiring an exit from the nurse led follow up (the primary outcome) and a discussion/review with a nephrologist and 1% of patients requiring a renal biopsy and immunosuppressive treatment. As with some other reports, we have identified older children presenting with HSP at the highest risk of renal involvement [19]. A stratified approach will allow focus on these individuals.

The second aim of this study was to revise and update our pathway using our data and the latest literature, to produce a follow up regimen that is evidence based and could be adapted for implementation in other institutions. Nurse led follow up has become an acceptable and more cost effective method for monitoring specific illnesses [20] and HSP screening could utilise the expertise of paediatric nurses. Within the UK, responsibility of HSP follow up is usually with the paediatric multidisciplinary team. The availability of appropriate blood pressure cuffs, trained phlebotomists, the expertise in alleviating parental anxiety, and a close working relationship with the multidisciplinary team are necessary to provide easy access to specialist care if needed.

**Figure 2:**
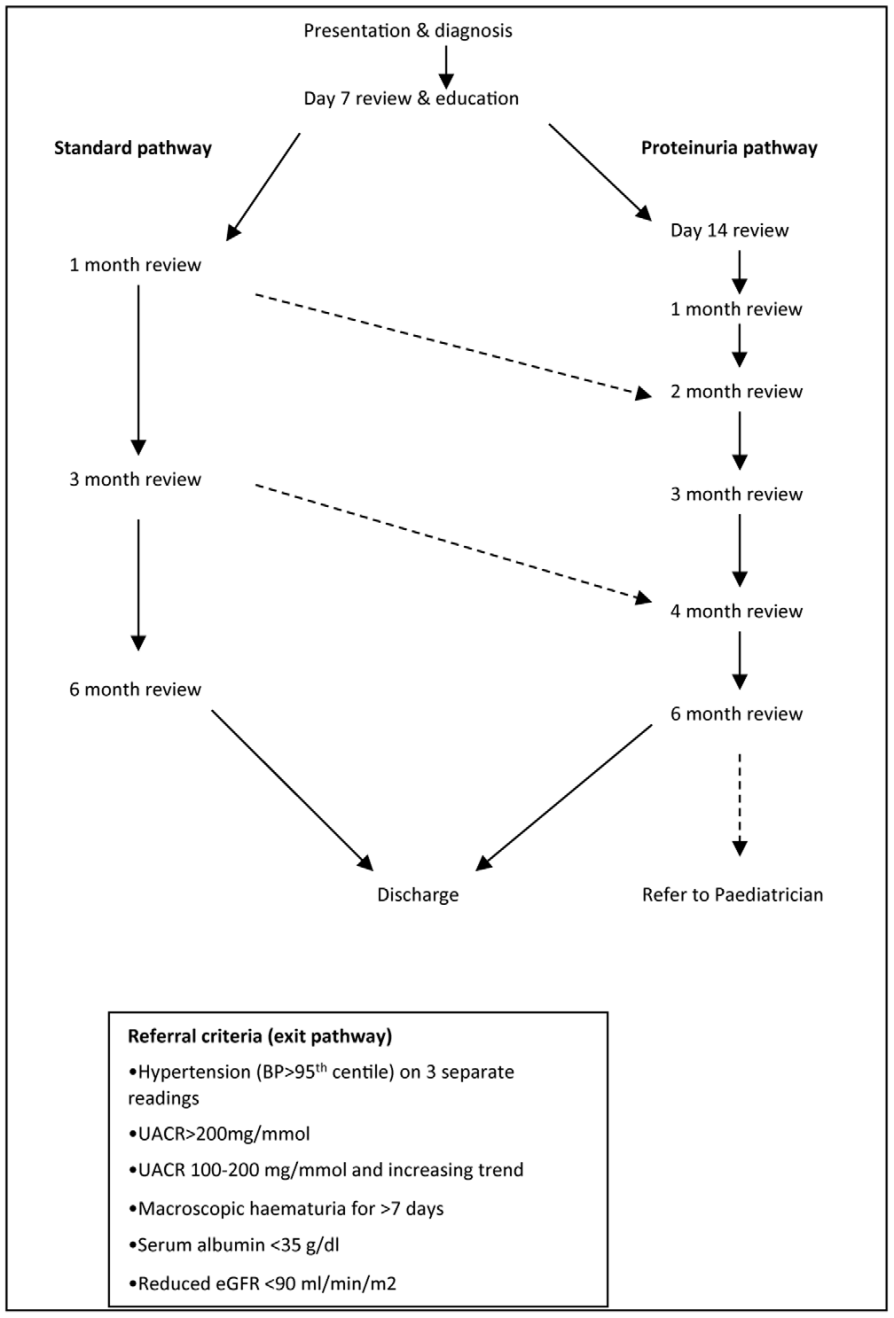
The Alder Hey Henoch Schonlein Purpura pathway - demonstrating follow up stratified according to the presence of proteinuria at the day 7 review.

The revised pathway, The Alder Hey Henoch Schonlein Purpura Pathway, is outlined in figure 2 and we shall now discuss the reasoning behind its formulation.

A meta-analysis by Narchi, confirmed that the current recommended length of follow up should be for 6 months regardless of the presenting clinical features or urinary findings [2], in our series all patients presented with proteinuria before 6 months after the initial HSP diagnosis. We have revised our pathway in agreement with this, to a 6-month monitoring period. Urine dipstick testing is easily accessible, can be performed with minimal training and is a good negative indicator in the absence of proteinuria [21]. It is generally accepted that moderate to severe proteinuria are clinical risk factors associated with a poor renal outcome [2], [18], [22]. The exact value of proteinuria during the acute period cannot predict the renal prognosis but the absence of proteinuria is known to be a good prognostic marker [3]. This is confirmed in our cohort where the acute urinalysis had an excellent negative predictive value, 97% of patients with a normal urinalysis would not require a renal referral. This finding will allow more accurate, individualised patient/parent counselling with regard to the likely renal consequences of HSP. We have used the urine dipstick as a first line measure of proteinuria in our pathway and a measure of stratifying follow up for these reasons. If the urine dipstick is positive, further quantification of proteinuria is required as specified in our primary investigations (table 2).

**Table 2:** Primary investigations for any case of proteinuria (urine dipstick ≥1+) and secondary investigations for patients meeting the predefined exit criteria, along with same day medical review.

**Primary investigations**
Urea & electrolytes
Creatinine
Serum albumin
Urine albumin:creatinine ratio
Urine microscopy
**Secondary investigations**
Anti streptococcal titre (ASOT)
Antinuclear antibody (ANA)
Double stranded DNA (dsDNA)
Anti-neutrophil cytoplasmic antibody (ANCA)
Complement profile - C3, C4
Full blood count
Clotting
Erythrocyte sedimentation rate (ESR)
C reactive protein (CRP)
Immunoglobulin profile
Renal Ultrasound

At day 7, all patients will begin the revised monitoring. This review is vital for provision of hand held records and parent education. At this point, patients are triaged into either a standard or more intensive (‘proteinuria’) follow up regimen. The revised pathway ensures patients with a normal urinalysis receive a less intensive follow up, but with a safeguard to convert onto the more intensive monitoring if proteinuria develops. In the revised pathway, decisions based on urinalysis should utilise early morning samples. Those who develop proteinuria in the first month are at the highest risk of renal complications, the risk of developing significant proteinuria after 1 month was extremely low in our series. Monitoring after this time point in patients with normal urinalysis is therefore less frequent until 6 months is complete.

Patients who have an abnormal urinalysis at day 7 will be triaged to the more intensive follow up with reviews fortnightly in the first month after diagnosis, then monthly until 4 months then a subsequent visit at 6 months. The intervals have been carefully selected using the knowledge that patients who develop nephrotic or nephritic features are at greatest risk of a poor renal outcome and that these features are likely to present within the first few months. At each visit if proteinuria is present primary investigations are required and the results will be assessed to see if the clinical features meet the specified exit criteria and warrant secondary investigations (table 2) and same day medical review. In either arm of the pathway if the urinalysis and blood pressure has remained or becomes normal children can be discharged at 6 months.

The exit criteria in the pathway, include hypertension, increased urine albumin:creatinine ratio, reduced serum albumin, reduced glomerular filtration rate and persistent macroscopic haematuria (figure 2). Hypertension has not clearly been linked to a poorer outcome in patients with HSP nephritis however it will require treatment and further investigation and so it is important to identify and act accordingly. In line with current best practice confirmation of hypertension should include measurement using a manual sphygmomanometer repeated on three separate occasions [2]. The rate of hypertension at diagnosis in our series should be interpreted with caution, repeat measurements over several days were not made, data was retrospectively reviewed and initial measurements utilised an oscillometric device. However we have demonstrated a rarity of hypertension during the monitoring period. Further studies specifically focused on evaluating hypertension, particularly during the acute inflammatory period in HSP, are required.

The presence of proteinuria is a very important exit criterion as it is the marker most commonly correlated with the renal prognosis. Within the exit criteria, we have used the urine albumin:creatinine ratio as a measure of proteinuria as it is sufficiently related to 24 hour protein levels and more convenient for paediatric practice [23]. The defined cut off values have been based on previous cohort data which identified patients with moderate to severe proteinuria at greater risk of poor renal outcome [22], [24] and so we require all patients with a UACR between 100–200 mg/mmol with an increasing trend and those with a UACR>200 mg/mmol to immediately exit from the pathway for further investigation and management in liaison with a paediatric nephrologist. Those patients with a UACR below these set values, whilst undeniably having significant proteinuria, continue to be monitored on the pathway as there is a high rate of spontaneous resolution (27 out of 35 on our proteinuria pathway resolved) and a lack of evidence demonstrating a poor renal outcome. Serum albumin levels <35 g/dL will assist with identifying nephrotic patients and require exit from the pathway and a medical review.

Reduced glomerular filtration rate is linked to a worse long-term renal prognosis [24], [25]. Early identification of impaired renal function is an important part of our pathway. Haematuria is commonly present in HSP nephritis and usually runs a benign course when isolated [24]. Macroscopic haematuria for more than 7 days in our cohort was rare and would require further investigation to explore differential diagnoses.

In summary the stated pathway exit criteria are relatively rare, require secondary investigation (table 2) and medical review. These features identify children who should be discussed with a nephrologist and considered for histological evaluation and immunosuppressive intervention. Future validation of the exit criteria is required and may provide a good opportunity for identifying patients for entry into future trials [26], [27].

HSP screening programs vary greatly across the country and around the world. Previously, Tizard was one of the first authors to describe a recommended pathway for the follow up of patients with HSP which triaged patients to additional investigations according to proteinuria, however this pathway still required frequent reviews for patients with normal urine findings [28]. We have attempted to balance screening for renal involvement against the inconvenience to the majority of patients who are at low risk of a poor renal outcome using our data and the best available evidence, whilst maintaining a six-month monitoring period in all patients due to the limitations of our study. In addition to producing a low cost and more convenient regimen, a standardised follow up would facilitate randomised controlled trials to assess the most appropriate therapy for this condition.

Limitations of this study include regionality, sample size and missing datasets. Although not statistically significant, there was a tendency for more renal involvement in case notes available for inclusion in this manuscript. Despite this, it has clearly highlighted the need for larger, multi-centre studies to improve our understanding of HSP. Importantly, future prospective, larger, representative studies would need to assess the strength of this revised nurse-led pathway. In addition, clinical trials are urgently needed to assess the effect of treatment interventions. A standardised approach to HSP and the development of national and international HSP registries will improve our understanding and management of this childhood vasculitis.

In summary, unlike all other complications of HSP, nephritis is typically asymptomatic and requires active screening. This study provides important follow up data on the natural course of HSP associated nephritis. Using these data, we have clinically evaluated, critically appraised and subsequently revised the evidence based monitoring regimen for patients with HSP, producing the Alder Hey Henoch Schonlein Purpura pathway. Following validation, implementation of this pathway could be achievable in a variety of settings, including some resource poor countries, and would facilitate future studies.

## Methods

### HSP pathway

Alder Hey Children's Hospital (UK) provides secondary care to children with HSP presenting to Accident and Emergency or via the family doctor, and tertiary care to children with renal involvement, either identified during monitoring or referred from other centres. A nurse led care pathway for HSP follow up was formulated based on best evidence and practice in 2004 [3], [11], [29], [30]. Children are diagnosed by the attending clinician according to the ACR classification criteria [31], following baseline investigations, and then referred to the nurse led pathway via an internal computerised system. Initial follow up review is within seven days of diagnosis (referred to throughout as ‘day 7 review’) and involves urinalysis for dipstick testing (Roche), blood pressure (BP) measurement using an oscillometric devise (Dinamap, Newport, USA) and parent education. Hand held records and written information are provided with hospital contact information and an open door policy. At initial review (day 7), children are triaged into a “standard” or “proteinuria” pathway according to the urine dipstick result. If a patient does not attend, a letter is sent asking the parents to arrange another appointment. A failure to reply, results in discharge and responsible consultant and family doctor being informed.

On the standard pathway (seen on day 7, day 14, 1 month, 3 month, 6 month, 12 month), each review consists of BP measurement and urinalysis. If proteinuria develops (defined as ≥1+) at any time point additional primary investigations are performed (table 2) and patients then transfer to the proteinuria pathway. Patients on the proteinuria pathway are seen more frequently (seen on day 7, day 14, 1 month, 2 month, 3 month, 4 month, 6 month, 12 month). At 12 months, patients with no urine or BP abnormalities are discharged. Those with proteinuria or haematuria are referred to a paediatrician. If a patient meets the predefined ‘exit criteria’ at any stage - hypertension, urine albumin:creatinine ratio (UACR) >200 mg/mmol, macroscopic haematuria >28 days, serum albumin <30 g/dl, reduced estimated glomerular filtration rate (GFR) <80 ml/min/m^2^ (using the Schwartz formula [32]) - they leave the nurse led pathway and receive a same day medical review with secondary investigations (table 2) and discussion with a paediatric nephrologist.

### Study Case Retrieval and Eligibility

Patients were identified using the hospital diagnostic coding system. Demographic and outcome data were recorded. All cases were included if they had undergone follow up on the specific pathway during the study period. Patients presenting with a second occurrence of HSP were treated in the same manner as those with a first episode of disease. Exclusion criteria included those patients without a diagnosis of HSP, patients with irretrievable pathway data and tertiary referrals for HSP nephritis already diagnosed in another centre (to ensure an unselected HSP cohort). To facilitate clinical data collection a standardised proforma was used.

*A priori* definition of hypertension and proteinuria/haematuria were used. Hypertension was defined as a systolic BP reading >95^th^ centile for the child's age, sex and height [33]. Significant proteinuria/haematuria was defined as ≥1+ on random urinalysis.

### Outcomes

The primary outcome for this study was the need to exit the nurse led pathway either due to persistent proteinuria or haematuria at 12 months or due to fulfilment of the earlier exit criteria, and thus prompting medical review at an earlier time point.

### Statistical analysis

Demographic data were analysed for eligible patients. Patients were described according to the presenting features, evolving renal features and renal outcome after 12 months follow up. Statistics Package for Social Sciences (SPSS Ltd, USA) version 18.0 was used. Probability (‘p’) values <0.05 were considered significant. Results are presented as median values (interquartile ranges). Positive and negative predictive values (confidence intervals) and specificity and sensitivity (confidence intervals) were calculated using the day 7 urinalysis as a screening tool to predict patients who will develop renal involvement and require a medical review.

### Ethical review

This study assessed current clinical practice and therefore, an ethical review was not necessary under UK National Health Service (NHS) research governance arrangements.
